# Activation of lysosomal iron triggers ferroptosis in cancer

**DOI:** 10.21203/rs.3.rs-4165774/v1

**Published:** 2024-04-08

**Authors:** Raphaël Rodriguez, Tatiana Cañeque, Leeroy Baron, Sebastian Müller, Alanis Carmona, Ludovic Colombeau, Antoine Versini, Marie Sabatier, Julio Sampaio, Eikan Mishima, Armel Picard-Bernes, Stéphanie Solier, Jiashuo Zheng, Bettina Proneth, Leishemba Thoidingjam, Christine Gaillet, Laurence Grimaud, Cameron Fraser, Krystina Szylo, Caroline Bonnet, Emmanuelle Charafe, Christophe Ginestier, Patricia Santofimia, Nelson Dusetti, Juan Iovanna, Antonio Sa Cunha, Gabriella Pittau, Pascal Hammel, Dimitri Tzanis, Sylvie Bonvalot, Sarah Watson, Brent Stockwell, Marcus Conrad, Jessalyn Ubellacker

**Affiliations:** Institut Curie, CNRS; Institut Curie; Institut Curie; Institut Curie, CNRS, INSERM, PSL Research University, Equipe Labellisée Ligue Contre le Cancer; Harvard T. H. Chan School of Public Health; Institut Curie; Institut Curie; Harvard T. H. Chan School of Public Health; Institut Curie; Institute of Metabolism and Cell Death, Molecular Targets & Therapeutics Center, Helmholtz Munich, Neuherberg, Germany; Institut Curie; Institut Curie; Helmholtz Zentrum München; Helmholtz Munich; Institut Curie; Institut Curie; ENS; Harvard University; Harvard T. H. Chan School of Public Health; Inserm; Inserm; Inserm; Inserm; Centre de Recherche en Cancérologie de Marseille, CRCM, Inserm, CNRS, Institut Paoli-Calmettes, Aix-Marseille Université, Marseille, France.; Centre de Recherche en cancerelogie de Marseille; APHP; APHP; APHP; Institut Curie; Institut Curie; Insitut Curie; Columbia University Irving Medical Center; Institute of Metabolism and Cell Death, Molecular Targets & Therapeutics Center, Helmholtz Munich, Neuherberg, Germany; Harvard T.H. Chan School of Public Health

## Abstract

Iron catalyses the oxidation of lipids in biological membranes and promotes a form of cell death referred to as ferroptosis^1–3^. Identifying where this chemistry takes place in the cell can inform the design of drugs capable of inducing or inhibiting ferroptosis in various disease-relevant settings. Whereas genetic approaches have revealed underlying mechanisms of lipid peroxide detoxification^1,4,5^, small molecules can provide unparalleled spatiotemporal control of the chemistry at work^6^. Here, we show that the ferroptosis inhibitor liproxstatin-1 (Lip-1) exerts a protective activity by inactivating iron in lysosomes. Based on this, we designed the bifunctional compound fentomycin that targets phospholipids at the plasma membrane and activates iron in lysosomes upon endocytosis, promoting oxidative degradation of phospholipids and ferroptosis. Fentomycin effectively kills primary sarcoma and pancreatic ductal adenocarcinoma cells. It acts as a lipolysis-targeting chimera (LIPTAC), preferentially targeting iron-rich CD44^high^ cell-subpopulations^7,8^ associated with the metastatic disease and drug resistance^9,10^. Furthermore, we demonstrate that fentomycin also depletes CD44^high^ cells *in vivo* and reduces intranodal tumour growth in an immunocompetent murine model of breast cancer metastasis. These data demonstrate that lysosomal iron triggers ferroptosis and that lysosomal iron redox chemistry can be exploited for therapeutic benefits.

## Introduction

Iron reacts with hydrogen peroxide to produce oxygen-centred radicals, initiating a chain reaction that leads to oxidised organic products, a process broadly defined as the Fenton reaction^11^. Chemically reactive lipids in biological membranes are ideal substrates for such reactions. Accumulation of damaged phospholipids can eventually cause loss of membrane integrity, altered organelle functions and release of components in the cell that can further promote cellular damage and cell death^12^. Ferroptosis has been shown to involve various organelles^13^ including peroxisomes^14^, mitochondria^15^, the endoplasmic reticulum (ER)^16^ and endolysosomes^17^. However, it is currently unclear whether individual organelles contribute to ferroptosis *via* altered signalling, metabolism and biosynthesis, or whether membrane lipids of these compartments are also direct substrates for iron-mediated oxidation of membrane lipids leading to cell death^18–21^. Therefore, where in the cell and the extent to which iron-mediated oxidation of membrane lipids promotes ferroptosis remain elusive.

## Results

### Lysosomal iron triggers ferroptosis

**Figure 1:**
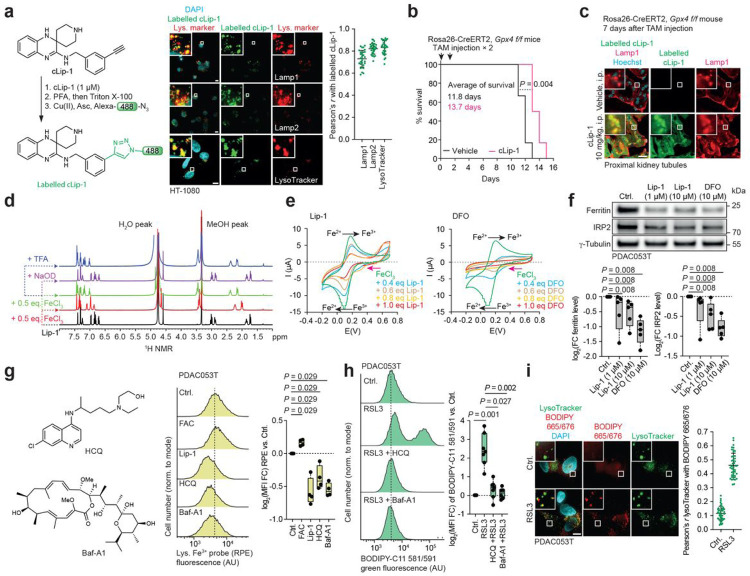
Lysosomal iron triggers ferroptosis. **a**, Experimental procedure for in-cell labelling of cLip-1 (1 μM, 1 h) by click chemistry and fluorescence microscopy of labelled cLip-1. Scale bars, 10 μm. At least 30 cells were quantified per condition. *n* = 3 independent experiments. Data are mean ± s.d. **b**, Kaplan-Meier survival curves of Rosa 26-CreERT2;*Gpx4* f/f mice treated with cLip-1 (10 mg/kg/day; intraperitoneal injection; *n* = 6 mice per group). Mantel–Cox log-rank test. **c**, Fluorescence microscopy of cLip-1 labelled in renal proximal tubules of a Rosa 26-CreERT2, *Gpx4* f/f mouse 7 days after tamoxifen injection. Scale bars, 10 μm. **d**, ^1^H NMR spectra of Lip-1 recorded at 310 K in methanol-*d_4_*. **e**, Cyclic voltammetry measurements towards reduction potentials (pink arrows) of an FeCl_3_ solution. Data recorded in the presence of Lip-1 or DFO. **f**, Representative western blots of iron homeostasis regulators in cells treated with iron chelators for 6 h (n = 5). FC, fold change. **g**, Molecular structure of lysosomal pH regulators and flow cytometry of the lysosomal Fe^3+^ probe RPE in cells treated for 1 h. AU, arbitrary unit. *n* = 4 independent experiments. FAC (100 μg/mL), Lip-1 (10 μM), HCQ (100 μM), Baf-A1 (75 nM). **h**, Flow cytometry of BODIPY-C11 581/591 in PDAC cells pre-treated with RSL3 for 7 h and treated with lysosomal pH regulators for 17 h. FC, fold change. Kruskal-Wallis test with Dunn’s post-test. RSL3 (200 nM), HCQ (10 μM), Baf-A1 (75 nM). **i**, Fluorescence microscopy of BODIPY 665/676 in PDAC cells treated for 1 h. Scale bars, 10 μm. At least 40 cells were quantified per condition. *n* = 3 independent experiments. Data are mean ± s.d. RSL3 (1 μM). f, g, Two-sided Mann–Whitney test. In all box plots in the main figures, boxes represent the interquartile range, centre lines represent medians and whiskers indicate the minimum and maximum values.

We set out to determine the subcellular sites of action of liproxstatin-1 (Lip-1), a small molecule that protects cells against ferroptosis induced by inactivation of oxidised lipid-detoxification systems, including the cystine/glutamate antiporter (SLC7A11/SLC3A2)/glutathione (GSH)/glutathione peroxidase 4 (GPX4) and NAD(P)H/ferroptosis suppressor protein 1 (FSP1)/quinone nodes^22^. In-cell labelling of an alkyne-containing synthetic analogue of Lip-1, we named cLip-1, in HT-1080 fibrosarcoma cells using click chemistry^6^ revealed lysosomal targeting using fluorescence microscopy (Fig. 1a **and Extended Data Fig. 1a,b**). *In vivo*, cLip-1 delayed death in mice undergoing acute renal failure as a result of genetic deletion of *Gpx4*^4^. Labelling cLip-1 in mouse tissues revealed its accumulation in the liver and kidney, co-localizing with a lysosomal marker in renal proximal tubules (Fig. 1b,c **and Extended Data Fig. 1c**). *In vitro*, cLip-1 prevented oxidation of membrane lipids and protected cells from genetic depletion of *Gpx4* or pharmacological inhibition of GPX4 with RSL3, essentially recapitulating the biological activity of Lip-1 (**Extended Data Fig. 1d-f**). These data validated cLip-1 as a suitable surrogate of Lip-1 to investigate ferroptosis. It is noteworthy that labelled cLip-1 was predominantly found to co-localize with a lysosomal marker at concentrations higher than the lowest effective dose, supporting the notion that Lip-1 exerts an anti-ferroptotic activity specifically in this organelle.

Iron is predominantly internalised by endocytosis in cancer cells^7,23^, regulating cell-state transitions and proliferation^7,24,25^. The observed accumulation of cLip-1 in lysosomes raised the prospect of ferroptosis inhibition by iron inactivation in this organelle, contrasting with a free radical-trapping activity^26^. Indeed, the iron chelator deferoxamine (DFO) has been shown to protect cells against erastin-induced ferroptosis^2^ and Lip-1 contains an iron-chelating o-phenyldiamine core^27^, further supporting this hypothesis. Nuclear magnetic resonance (NMR) spectroscopy and visual inspection indicated that Lip-1 forms complexes with iron(III) that are stable under acidic conditions, such as those found in lysosomes (e.g. pH<5), but which dissociate at higher pH (pH>12) (Fig. 1d **and Extended Data Fig. 2a**). Cyclic voltammetry indicated that Lip-1 and DFO impair iron redox properties (Fig. 1e). As controls, we investigated two other synthetic analogues of Lip-1, namely metcLip-1 and alcLip-1. In metcLip-1, aromatic amines are methylated, adversely impacting on radical-trapping capacity^26^. In contrast, alcLip-1 is an aliphatic analogue characterised by higher pKa of amines. It is expected to be already protonated at physiological pH and thus to exhibit a reduced propensity to accumulate in lysosomes and to form tight complexes with iron (**Extended Data Fig. 2b**). Labelling of metcLip-1 in cells revealed a similar staining pattern as that of labelled cLip-1, showing co-localisation with a lysosomal marker, whereas a weaker fluorescence of labelled alcLip-1 was detected (**Extended Data Fig. 2c**). Cyclic voltammetry further indicated that metcLip-1 retained some level of redox inactivation capacity towards iron, whereas alcLip-1 did not exhibit any measurable effect (**Extended Data Fig. 2d**). Consistently, metcLip-1 protected cells against RSL3-induced oxidation of membrane lipids and cell death, although to a lesser extent than Lip-1. This reflected the reduced capacity of this analogue to inactivate iron, presumably due to steric hindrance of the methyl substituents. In comparison, alcLip-1 was biologically inactive in this context (**Extended Data Fig. 2e,f**). In-cell labelling of an alkyne-containing derivative of DFO (cDFO)^7,8^ revealed nuclear and lysosomal fluorescence signals (**Extended Data Fig. 2g**). Both DFO and Lip-1 induced degradation of the iron storage protein ferritin and iron regulatory protein 2 (IRP2) in primary human pancreatic ductal adenocarcinoma (PDAC) cells, indicating that upon binding to lysosomal iron, these compounds deplete the available cellular iron pool^28^, providing a rationale for their toxicity at high concentrations (Fig. 1f). Treatments with hydroxychloroquine (HCQ) or bafilomycin-A1 (Baf-A1), which raise the lysosomal pH and prevent iron(III) unloading from its endocytic carriers, led to reduced pools of free lysosomal iron(III) and protected cells against RSL3-induced oxidation of membrane lipids. Baf-A1 also protected cells against RSL3-induced death (Fig. 1g,h **and Extended Data Fig. 2h**). Upon treatment with RSL3 for 1 h, membrane lipid oxidation was predominantly detected in lysosomes according to BODIPY fluorescence. By contrast, treatment with RSL3 for 4 h led to a staining indicative of oxidised membrane lipids that co-localised mainly with a fluorescently-labelled biological marker of the ER (Fig. 1i **and Extended Data Fig. 3a,b**). Our data suggest that initiation of the radical chain reaction takes place early in the lysosomal compartment, where redox-active iron(II) can be found^18^. This radical chain reaction then propagates to membrane lipids of other proximal organelles including the ER. In support to this, treating cells with well-established ferroptosis inducers led to a depletion of glutathione (GSH) and an increase of hydroxyl radicals in lysosomes (**Extended Data Fig. 3c-f**). Together, these data illuminate the central role of lysosomal iron as a ferroptosis trigger.

### Development of a lipolysis-targeting chimera

**Figure 2:**
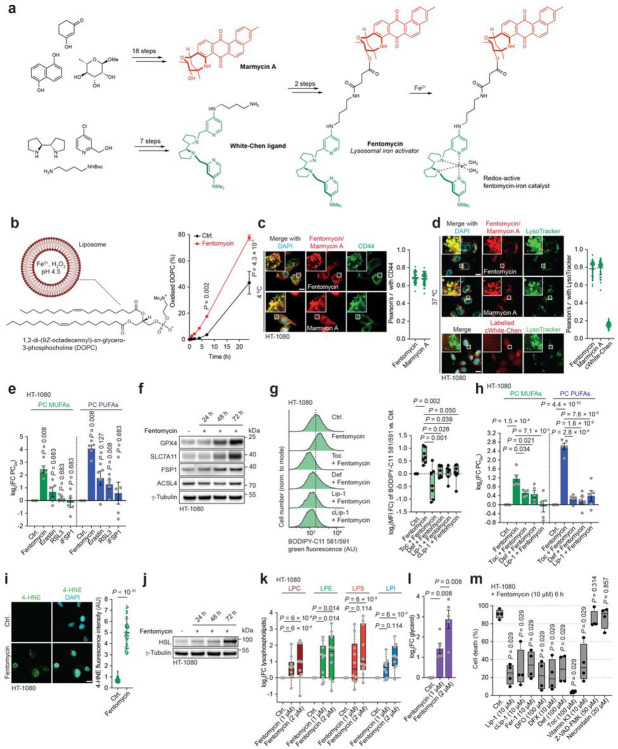
Development of a lipolysis-targeting chimera. **a**, Chemical synthesis of fentomycin and production of the redox-active iron catalyst *in situ*. **b**, Oxidation rate of DOPC-forming liposomes in the presence of fentomycin. 2-way ANOVA. Mean values ± s.e.m. **c**, Fluorescence microscopy of fentomycin, marmycin A and CD44 in cells treated at 4 °C. 45 cells were quantified per condition. *n* = 3 independent experiments. Data are mean ± s.d. **d**, Fluorescence microscopy of fentomycin, marmycin A and cWhite-Chen ligand labelled *in situ* in cells treated at 37 °C. 45 cells were quantified per condition. *n* = 3 independent experiments. Data are mean ± s.d. **e**, Quantitative mass spectrometry-based lipidomics of oxidised phospholipids. *n* = 5 independent experiments. **f**, Representative western blot of ferroptosis regulators. *n* = 3 independent experiments. **g**, Flow cytometry of BODIPY-C11 581/591 in cells treated for 24 h. *n* = 7 independent experiments. Kruskal-Wallis test with Dunn’s post-test. **h**, Quantitative mass spectrometry-based lipidomics analysis of oxidised phospholipids in cells treated for 24 h. *n* = 5 independent experiments. **i**, Fluorescence of 4-HNE treated with Fentomycin for 1 h. 50 cells were quantified per condition. Two-sided unpaired *t*-test. Data are mean ± s.d. **j**, Representative western blot of lipases. *n* = 3 independent experiments. **k**, Quantitative mass spectrometry-based lipidomics of lysophospholipids in cells treated for 24 h. *n* = 10 independent experiments. **l**, Quantification of glycerol in cells treated for 24 h. Mean values ± s.e.m. **m**, LDH release from cells pre-treated for 2 h with inhibitors, then treated with 10 μM fentomycin for 6 h. *n* = 4 independent experiments. Fer-1, ferrostatin-1; Toc (tocopherol), DFO, deferoxamine; DFX, deferasirox; Def, deferiprone. The following concentrations were used unless stated otherwise: Fentomycin (1 μM), erastin (10 μM), RSL3 (100 nM), iFSP1 (10 μM), Toc (100 μM), Def (100 μM), Lip-1 (1 μM) and cLip-1 (1 μM). Scale bars, 10 μm. **e, h, k-m**, Two-sided Mann–Whitney test.

Therapy-resistant cancer cells have been shown to be vulnerable to ferroptosis^18,29–31^. These cells can overexpress the cancer stem cell marker and iron transporter CD44, a membrane glycoprotein associated with tumourigenesis and cancer metastasis^9,32–34^. By doing so, these cells upregulate iron endocytosis to promote the activity of iron-dependent demethylases, enabling specific transcriptional programs underlying cell-state transitions and acquisition of a drug-tolerant persister cancer cell phenotype^7^. Thus, lysosomal iron has emerged as a tractable druggable target to promote ferroptosis in a cell state-dependent manner^10^. With this in mind, and the knowledge that lysosomal iron can trigger oxidation of membrane lipids, we rationally designed a small molecule to target lipids at the plasma membrane, which upon endocytosis activates iron(II) in lysosomes and promotes Fenton-like chemistry, exploiting membrane lipids as substrates for oxidation. To this end, we designed a chimera of the fluorescent lipophilic natural product marmycin A and the synthetic White-Chen ligand, which we named fentomycin (Fig. 2a **and Extended Data Fig. 4a-e**). Marmycin A has been shown to accumulate at the plasma membrane of cells and to be internalised by endocytosis^35^, whereas the White-Chen ligand is commonly used in chemical synthesis to oxidize organic substrates by activating iron(II)^36–38^. In the presence of hydrogen peroxide and under mild acidic aqueous conditions, such as those found in lysosomes, the White-Chen iron catalyst is thought to form a reactive iron-oxo intermediate, which, like hydroxyl and hydroperoxyl radicals, is able to abstract a hydrogen atom from organic substrates, including fully saturated and least reactive ones, to produce reactive carbon-centred radicals, leading to oxidation products^39,40^. Fentomycin is therefore reminiscent of bifunctional molecular glues able to induce proximity *de novo* to manipulate protein function or to induce degradation^41–43^. We hypothesised that by using the abundant reactive iron(II) in the drug-tolerant cell state, such a chimera would form an active catalyst *in situ* susceptible to promote oxidative degradation of proximal membrane lipids in lysosomes, ultimately triggering ferroptosis.

In a cell-free system, fentomycin accelerated the oxidation of a liposome-forming unsaturated phospholipid under experimental conditions comparable to that found in lysosomes including acidic pH, the presence of hydrogen peroxide and a water soluble iron(II) salt (Fig. 2b). The intrinsic fluorescence of fentomycin revealed its localisation at the plasma membrane together with CD44, when experiments were conducted at low temperature to reduce endocytic flux (Fig. 2c), recapitulating the photophysical properties of the parental marmycin A. In contrast, at a physiological temperature, fentomycin and marmycin A were found to target the lysosomal compartment, whereas chemical labelling of an alkyne-containing White-Chen (cWhite-Chen) ligand revealed a weak pan-cellular staining (Fig. 2d **and Extended Data Fig. 4f**).

Fentomycin induced the oxidation of membrane lipids in HT-1080 cells, comparing favourably with well-established ferroptosis inducers, as shown by mass spectrometry-based lipidomics (Fig. 2e, **Extended Data Fig. 5a and Supplementary Table 1**). Furthermore, fentomycin induced oxidation of membrane lipids and altered cell viability in a series of human and murine PDAC cell lines and primary cells, whereas the biological activities of marmycin A and cWhite-Chen ligand were marginal (**Extended Data Fig. 5b,c**). Consistent with the oxidation of membrane lipids, sublethal doses of fentomycin led to an increase of the ferroptosis gatekeepers GPX4 and SLC7A11 in HT-1080 cells (Fig. 2f), and membrane lipid oxidation was prevented by the lipophilic antioxidant tocopherol (Toc), the iron chelator deferiprone (Def) and Lip-1 (Fig. 2g,h, **Extended Data Fig. 5d and Supplementary Table 2**). Fentomycin further induced the production of 4-hydroxynonenal (4-HNE) (Fig. 2i), which is characteristic of peroxidation and breakdown of fatty acid chains^44^. Interestingly, 4-HNE can induce cellular damage and its production is a hallmark of ferroptosis^45^. Longer treatment of cells with fentomycin led to the upregulation of hormone sensitive lipase (HSL) and an increase of lysophospholipids and glycerol (Fig. 2j–l **and Supplementary Table 3**), which suggests that oxidised phospholipids trigger a membrane remodelling response^46^. Finally, fentomycin-induced cell death was antagonised by well-established ferroptosis inhibitors, which included iron chelators and antioxidants, but not by apoptosis or necroptosis inhibitors (Fig. 2m **and Extended Data Fig. 5e,f**). It is noteworthy that fentomycin exhibits a residual toxicity that ferroptosis inhibitors cannot fully overcome. Together, these data indicate that pharmacological activation of lysosomal iron can trigger ferroptosis, with fentomycin acting as a LIPolysis-TArgeting Chimera (LIPTAC).

### Fentomycin induces ferroptosis in cancer

We next evaluated the effect of lysosomal iron activation in disease-relevant models. To this end, we investigated the iron content of primary tumour tissues of distinct cancer types, including human PDAC, various human sarcoma subtypes and a murine model of spontaneous breast cancer metastasis. These were chosen for their refractory nature to standard-of-care treatments and capacity to form metastases, contributing to poor clinical outcomes. Furthermore, these indications have been shown to be vulnerable to ferroptosis^18,20,29,30,47^.

**Figure 3:**
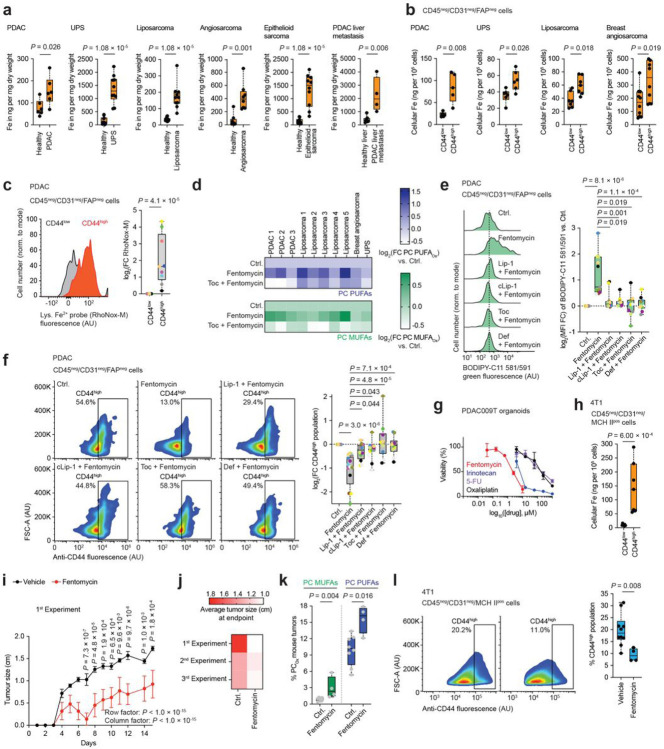
Pharmacological activation of lysosomal iron induces ferroptosis in drug-tolerant cancer cells. **a**, ICP-MS of iron in human healthy and cancer tissues. *n* = 4–10 technical replicates. **b**, ICP-MS of iron in cancer cells of dissociated human tumors. *n* = 5–10 technical replicates. **c**, Flow cytometry of the lysosomal Fe^2+^ turn-on probe RhoNox-M in cancer cells of dissociated human PDAC. *n* = 9 patients. **d**, Quantitative mass spectrometry-based lipidomics of oxidised phospholipids in dissociated human tumors pre-treated with Toc for 2 h, then co-treated with fentomycin for 24 h. *n* = 10 patients. **e**, Flow cytometry of BODIPY-C11 581/591 in cancer cells of dissociated human PDAC pre-treated with ferroptosis inhibitors for 2 h and then co-treated with fentomycin for 24 h. *n* = 11 patients. **f**, Flow cytometry of CD44 in cancer cells of dissociated human PDAC pre-treated with ferroptosis inhibitors for 2 h and then co-treated with fentomycin for 24 h. *n* = 13 patients. **g**, Cell viability in human PDAC-derived organoid treated with fentomycin or standard-of-care chemotherapy for 72 h. **h**, ICP-MS of iron in mouse 4T1 breast tumour cells. *n* = 4–8 technical replicates. **i**, Tumour growth in 4T1-tumour bearing mice treated with fentomycin (0.003 mg per animal every-other-day). Vehicle: *n* = 10 mice, fentomycin: *n* = 5 mice. 2-way ANOVA. Mean values ± s.e.m. **j**, Size of tumours in 4T1-tumour-bearing mice of *n* = 3 independent experiments. 1^st^ experiment vehicle: *n* = 10 mice, fentomycin: *n* = 5 mice; 2^nd^ experiment vehicle: *n* = 10 mice, fentomycin: *n* = 10 mice; 3^rd^ experiment vehicle: *n* = 5 mice, fentomycin: *n* = 5 mice. **k**, Quantitative mass spectrometry-based lipidomics of oxidised phospholipids in 4T1 tumours treated *in vivo* for 15 days. *n* = 4–8 mice. **l**, Flow cytometry of CD44 in dissociated 4T1 tumour cancer cells. The following concentrations were used unless stated otherwise: Fentomycin (1 μM), Toc (100 μM), Def (100 μM), Lip-1 (1 μM) and cLip-1 (1 μM). **a-c, h, k, l**, Two-sided Mann–Whitney test. **e, f**, Kruskal-Wallis test with Dunn’s post-test. **c, e, f**, each coloured dot represents a tumour of an individual patient for a given panel.

Inductively coupled plasma-mass spectrometry (ICP-MS) showed the total iron content to be higher in cancer compared to adjacent non-cancerous tissues of the same patients (Fig. 3a **and Extended Data Fig. 6a**), and the cellular iron load was found to be higher in subpopulations of cancer cells overexpressing CD44 (Fig. 3b). Studying cells from freshly dissociated human primary PDAC and sarcoma tissues showed higher levels of redox-active lysosomal iron(II) in the CD44^high^ subpopulations of cancer cells compared to their CD44^low^ counterparts (Fig. 3c **and Extended Data Fig. 6b**). This was consistent with prior findings that showed that CD44 mediates iron endocytosis in cancer cells acquiring a drug-tolerant persister phenotype^7^. In cells obtained from freshly dissociated human primary tumours, fentomycin induced oxidation and lipolysis of membrane lipids, an effect that was antagonised by ferroptosis inhibitors (Fig. 3d,e, **Extended Data Fig. 6c,d and Supplementary Table 4**). Remarkably, fentomycin also reduced the number of CD44^high^ cells in PDAC and undifferentiated pleomorphic sarcoma (UPS) and this was also antagonised by ferroptosis inhibitors (Fig. 3f **and Extended Data Fig. 6e,f**). In primary PDAC cells and human PDAC-derived organoids, fentomycin exhibited a more pronounced effect on cell viability compared to standard-of-care drugs, including irinotecan, 5-FU and oxaliplatin (Fig. 3g and **Extended Data Fig. 6g,h**).

Regional lymph node cancer lesions are important predictors of distant metastases and mortality. Recently, it has been shown that lymph protects metastasizing cells from ferroptosis^48^. ICP-MS showed lower total iron levels in lymph fluids compared to blood and serum (**Extended Data Fig. 7a**). Thus, we evaluated the effect of fentomycin on the viability of cancer cells directly in the lymphatics. To this end, we used the 4T1 immunocompetent murine model of spontaneous triple negative breast cancer metastases in Balb/c mice. Quantifying iron in cells isolated from intranodal 4T1 tumours showed a higher iron load in CD44^high^ cancer cells compared to the CD44^low^ subpopulations (Fig. 3h). These CD44^high^ cells also exhibited a higher iron(II)-redox activity in lysosomes and fentomycin altered cell viability *in vitro*, which was antagonised by Lip-1 (**Extended Data Fig. 7b,c**). Treating mice bearing intranodal 4T1 tumours with fentomycin by intranodal administration every-other-day led to an inhibition of tumour growth (Fig. 3i,j **and Extended Data Fig. 7d,e**). Further analyses of residual tumours indicated that fentomycin induced oxidation of membrane lipids and production of lysophospholipids, exhibiting its activity preferentially against CD44^high^ over CD44^low^ cancer cell subpopulations (Fig. 3k,l, **Extended Data Fig. 7f,g and Supplementary Table 5**). Together, these data support the idea that fentomycin elicits ferroptosis *in vivo* by exploiting the higher abundance of lysosomal iron(II) in CD44^high^ cell subpopulations.

## Discussion

**Figure 4:**
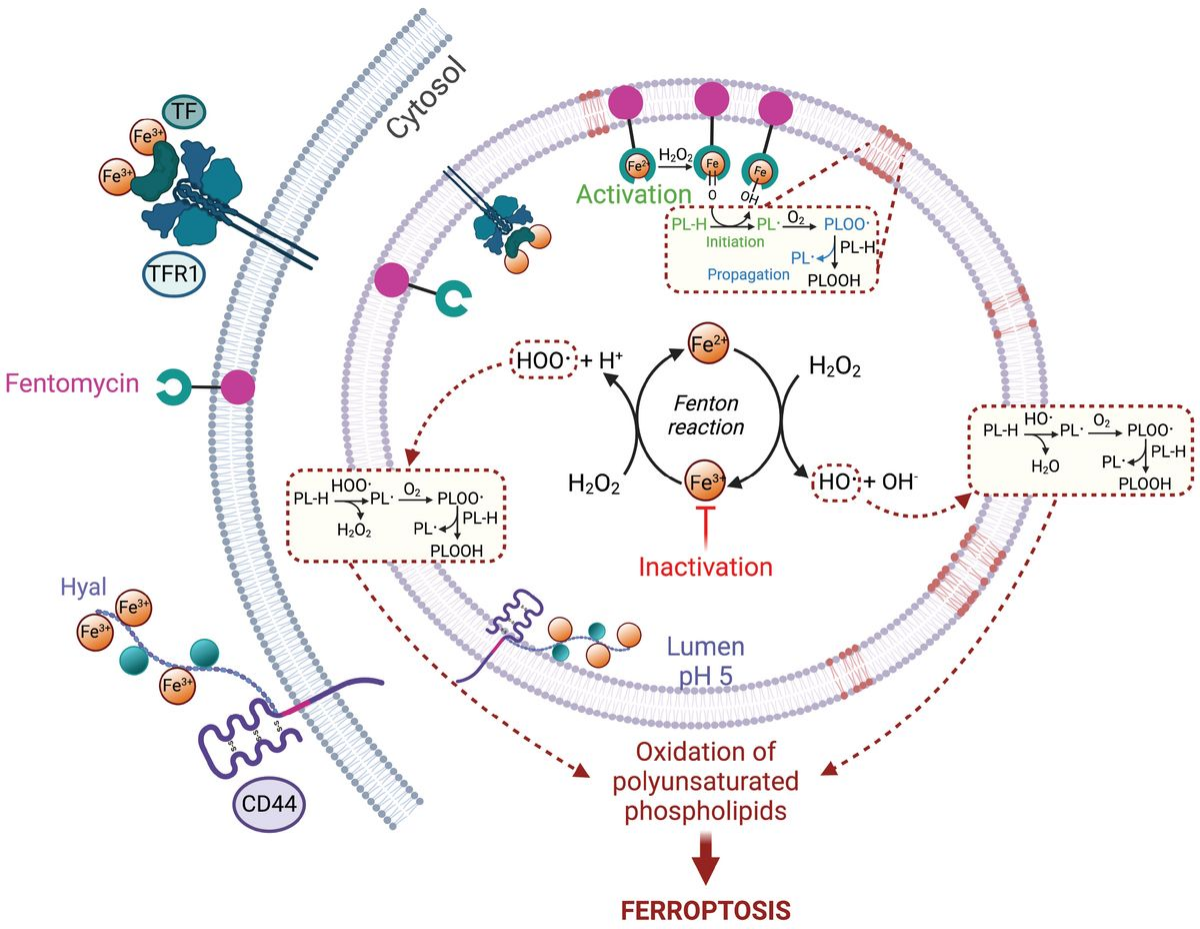
Mechanisms of ferroptosis initiation and blockade. Iron is internalised by endocytosis. Lysosomal iron catalyses the production of oxygen-centred radicals from hydroperoxides under acidic conditions. These radicals can abstract a hydrogen from reactive phospholipids to produce carbon-centred radicals leading to oxidation products and ferroptosis. Inactivation of lysosomal iron protects cells against iron-redox chemistry. Activation of lysosomal iron triggers oxidation of membrane lipids and ferroptosis.

Ferroptosis is a form of cell death resulting from the uncontrolled oxidation of membrane lipids. Whether this process is enzymatically driven, and where it is triggered in the cell had remained unclear. Iron can react with hydroperoxides to initiate or propagate a radical chain reaction independently of enzymes. The lysosomal compartment is a key regulator of cellular iron homeostasis^7,28,49^. Its acidic nature together with the presence of reactive iron and hydrogen peroxide provide the ideal chemical environment to catalyse the oxidation of membrane phospholipids (Fig. 4). Specific cancer cell subpopulations upregulate the iron transporter CD44 to promote oxidative demethylation of repressive chromatin marks and unlock the expression of genes involved in cancer progression^7^. Thus, while higher iron levels enable these cells to acquire a drug-tolerant profile, this also inexorably confers vulnerability to ferroptosis^10^. The fact that CD44 also marks development, self-renewal, wound healing and immune cell activation raises a putative contribution of lysosomal iron in promoting ferroptosis in these biological settings. Enhancing the redox activity of lysosomal iron by means of genetic intervention to eradicate cancer cells is challenging. Here, we have developed a lipolysis-targeting chimera that takes advantage of the higher iron load of specific cancer cell subpopulations to induce ferroptosis by activating lysosomal iron(II). We demonstrate that manipulating the redox activity of lysosomal iron provides control over membrane lipid oxidation, supporting the contention that lysosomal iron is a trigger of ferroptosis. Fentomycin exhibits a unique chemotype to investigate ferroptosis and provides a new paradigm to target drug-tolerant persister cancer cells^50^.

## Methods

### Ethics statement

Fresh tumour samples were obtained from patients undergoing surgery at Institut Curie and Paul Brousse hospitals. All patients provided written informed consent for use of tumour samples. The study was approved by institutional regulatory boards (DATA190160). All cLip-1 *in vivo* experiments were performed in compliance with the German Animal Welfare Law and have been approved by the Institutional Committee on Animal Experimentation and the Government of Upper Bavaria (approved no. ROB-55.2-2532.Vet_02-18-13).All intranodal injection mouse experiments complied with all relevant ethical regulations and were performed according to protocols approved by the Institutional Animal Care and Use Committee at Harvard T.H. Chan School of Public Health (protocol IS00003460). For mouse lymph and blood collection, animal experiments were performed in accordance with the European Community guiding in the care and use of animals.Animal experiments were performed in agreement with the French Guidelines for animal handling and approved by local ethics committee (Agreement no. 16487-2018082108541206 v3).

#### Leadcontact.

Further information and requests for resources and reagents should be directed to the lead contact Raphaël Rodriguez (raphael.rodriguez@curie.fr).

#### Materialsavailability.

Please contact the lead author Raphaël Rodriguez for in-house reagents, which can be made available under a material transfer agreement with Institut Curie.

#### Data availability.

Lipidomics data are presented in Supplementary Tables 1–5.

#### Chemicalsynthesis.

Starting materials were purchased at the highest commercial quality and used without further purification, unless otherwise stated. Anhydrous solvents were obtained by passing the degassed solvents through molecular sieves and activated alumina columns. Reactions were monitored by thin layer chromatography using aluminium plates coated with silica gel or neutral aluminium oxide neutral from Merck (60 F_254_). TLC plates were visualised by UV or by treatment with a ninhydrin, CAM or potassium permanganate solutions and heating. Reaction products were purified by flash column chromatography on silica gel 60 (230–400 mesh, Macherey Nagel) or aluminium oxide (activated neutral, Sigma-Aldrich), by Combiflash Rf, or by preparative HPLC Quaternary Gradient 2545 equipped with a Photodiode Array detector (Waters) fitted with a reverse phase column (XBridge BEH C18 OBD Prep column 5 μm 30 × 150 mm). NMR spectroscopy was performed on Bruker 300, 400 or 500 MHz instruments. Spectra were run in methanol-*d_4_*, dimethylsulfoxide-*d_6_*, methylene chloride-*d_2_* or chloroform-*d*, at 298 K. ^1^H chemical shifts δ are expressed in ppm using the residual non-deuterated solvent as internal standard and the coupling constants *J* are specified in Hz. The following abbreviations are used: bs, broad singlet; s, singlet; d, doublet; dd, doublet of doublets; ddd, doublet of doublet of doublets; dt, doublet of triplets; dq, doublet of quadruplets; q, quadruplet; t, triplet; quint., quintet; m, multiplet. ^13^C chemical shifts δ are expressed in ppm using the residual non-deuterated solvent as internal standard. The purity of final compounds was determined to be >98% by UPLC-MS. Low-resolution mass spectra (LRMS) were recorded on a Waters Acquity H-class equipped with a Photodiode array detector and SQ Detector 2 (UPLC-MS) fitted with a reverse phase column (Acquity UPLC BEH C18 1.7 μm, 2.1×50 mm). HRMS were recorded on a Thermo Scientific Q-Exactive Plus equipped with a Robotic TriVersa NanoMate Advion. Procedures for the synthesis of small molecules are detailed in the Supplementary Information.

#### NMR of Lip-1-iron(III) complexes:

^1^H NMR spectra were recorded on a 500 MHz Bruker spectrometer at 310 K, and chemical shifts δ are expressed in ppm using the residual non-deuterated solvent signals as internal standard. *General procedure:* From 0 to 1 equivalent of FeCl_3_ (Alfa Aesar, 12357, lot E23Z042), portions of 0.5 equivalent of a solution of FeCl_3_ in methanol-*d_4_* were added up to a solution of 1 equivalent of liproxstatin-1 (Lip-1, Sigma-Aldrich, SμL1414, lot 0000152075) in methanol-*d_4_* into an NMR tube and NMR spectra were recorded. Then a drop of trifluoroacetic acid (TFA, Sigma-Aldrich, T6508) or sodium deuteroxide (NaOD, Eurisotop, D076Y) was added. 0.94 mg of Lip-1 were dissolved in 600 μL of methanol-*d_4_*. From 0 to 1 equivalent of FeCl_3_, portions of 3.0 μL of 92 mM solution of FeCl_3_ were added.

#### Cyclic voltammetry.

Cyclic voltammetry^51^ experiments were performed with a three-electrode cell. A saturated calomel electrode (SCE) was used as reference, a steady glassy carbon (GC) electrode of diameter 3 mm was selected as working electrode and a platinum wire as counter-electrode. All cyclic voltammograms were recorded at room temperature with a μ-autolab III from Metrohm using Nova software with a scan rate of 2 V/s. MeCN and MeOH were used in HPLC grade (Carlo Erba). For all experiments we used a 0.1 M *n*Bu_4_NBF_4_ in MeCN (32.9 mg/μL stock solution). 1 mM FeCl_3_ solutions were prepared with 50 μL of 20 mM FeCl_3_ solution in milliQ water and 950 μL of MeCN. Then, portions of 10 μL (0.2 eq.) of 20 mM stock solution of Lip-1 or analogues (solubilised in MeCN or MeOH) were added until 1.0 eq. was reached. Above 1.0 eq., 50 μL (1.0 eq.) of 20 mM stock solution of the analogues were added. After each addition, the solution was stirred for a few seconds and voltammograms were recorded.

#### Complexation studies in solution.

100 mM stock solutions of DFO mesylate salt (Sigma-Aldrich, D9533), FeCl_3_ or Lip-1 were dissolved in water for DFO and FeCl_3_, and MeCN for Lip-1. For dilution of each compound, 15 μL of these stock solutions were added to 135 μL of MeCN to reach a concentration of 10 mM in each vial. For mixture of compounds, 15 μL of each compound were added to 120 μL of MeCN to reach a concentration of 10 mM. miliQ water and HPLC grade MeCN (Carlo Erba) were used.

#### Liposome preparation.

Liposomal structures were prepared using the traditional lipid film hydration method: 100 μL of a stock solution (1mg/μL chloroform) of 18:1 (Δ9-cis) PC (DOPC, Avanti Polar Lipids) were dissolved in 400 μL of chloroform and transferred into a round-bottom flask. The organic solvent was removed under reduced pressure in a rotary evaporator for 15 min at 200 rpm at 37 °C in a water bath. Afterwards, the lipid film was dried with a vacuum pump overnight. Then was hydrated with 1 μL of 0.1 mM sodium acetate buffer (pH 4.5) and vortexed every 5 min for 20 min. Liposomes were extruded by passing the suspension through 2 polycarbonate membranes (pore size 0.2 mm) 20 times.

#### Lipid oxidation in vitro.

Control experiment: 200 μL of the liposome solution were added into an Eppendorf tube and heated at 37 °C with agitation at 800 rpm. Then 5 μL of an aq. solution of Fe(OTf)_2_ (1.4 mg/1.5 μL) and 13 μL of 0.1 mM acetate buffer (pH 4.5) were added. At t = 0 min 13 μL of an aq. solution of H_2_O_2_ (10 μL H_2_O_2_ (30%)/1 μL) were added. Fentomycin experiment: 200 μL of the liposome solution were added into an Eppendorf tube and heated at 37 °C with 800 rpm. Then 13 μL of a solution 1 mM of fentomycin in DMSO and 5 μL of an aq. solution of Fe(OTf)_2_ (1.4 mg/1.5 μL) were added. At t = 0 min 13 μL of an aq. solution of H_2_O_2_ (10 μL H_2_O_2_ (30%)/1 μL) were added. The kinetic process of DOPC oxidation was recorded with a QExactive mass spectrometer (Thermo Fisher Scientific) equipped with a TriVersa NanoMate ion source (Advion Biosciences). Samples were injected at 0.5 h, 1 h, 2 h, 3 h, 4 h, 7 h and 24 h reaction time.

#### Antibodies.

Antibodies are annotated below as follows. WB, western blot; FCy, flow cytometry; FM, fluorescence microscopy. Hu, used for human samples. Ms, used for mouse samples. Dilutions are indicated. Any antibody validation by manufacturer is indicated and can be found on the manufacturers’ websites. Our antibody validation knockdown (KD) and/or KO strategies as described here for relevant antibodies is indicated. Primary antibodies: 4-Hydroxynonenal (4-HNE) (Abcam, ab48506, lot 1062274–2, FM, 1:200, Hu), ACSL4 (Santa Cruz Biotechnology, sc-271800, clone A-5, lot I1222, WB 1:1000, Hu, siRNA validated by us), AIFM2/FSP1 (Merck, MABC1638, clone 6D8–11, lot Q3745998, WB, 1:500, Hu, siRNA validated by us), Catalase (Cell Signaling, 12980T, clone D4P7B, lot 3, FM, 1:200, Hu), CD3-BV510 (BioLegend, 317332, clone OKT3, lot B263750, FCy, 1:100, Hu), CD31-PE-Cy7 (BioLegend, 303118, clone WM59, lot B276836, FCy, 1:100, Hu), CD31-BV605 (BioLegend, 303122, clone WM59, lot 331683, FCy, 1:100, Hu), CD31-BV605 (BioLegend, 102427, clone 390, lot B375532, FCy, 1:100, Ms), CD44-AF647 (Novus Biologicals, NB500–481AF647, clone MEM-263, lot D145771, FCy, 1:100, Hu), CD44-AF647 (BioLegend, 103018, clone IM7, lot B317762, FCy, 1:100, Ms), CD45-BV785 (BioLegend, 304048, clone HI30, lot B339809, FCy, 1:100, Hu), CD45-BV510 (BioLegend, 368526, clone 2D1, lot B373428, FCy, 1:100, Hu), CD45-BV510 (BioLegend, 103138, clone 30-F11, lot B386738, FCy, 1:100, Ms), CD163-PerCP/Cyanine5.5 (BioLegend, 326512, clone RM3/1, lot B291202, FCy, 1:100, Hu), Cytochrome *c* (Cell Signaling, 12963S, clone 6H2.B4, lot 2, FM, 1:200, Hu), EEA1 (Abcam, ab70521, clone 1G11, lot GR315680–1, FM, 1:200, Hu, validated in ICC/IF by manufacturer), FAP-AF700 (R&D Systems, FAB3715N, clone 427819, lot AEVI020011, FCy, 1:100, Hu), FAP-AF750 (Novus Biologicals, FAP3715S, lot 1718688, FCy, 1:100, Hu), Ferritin (Abcam, ab75973, clone EPR300AY, lot 10136442–29, WB, 1:1000, Hu, validated in WB by manufacturer), GPX4 (Abcam, ab125066, clone EPNCIR144, lot GR3369574–4, WB, 1:2000, Hu, KO validated by manufacturer), HSL (Cell Signaling, 18381T, clone D6W5S, lot 1, WB, 1:1000, Hu), IRP2 (Novus Biologicals, NB 100–1798, lot D-3, WB, 1:1000, Hu, validated by manufacturer), Lamp1 (Cell Signaling, 9091S, clone D2D11, lot 7, FM, 1:200, Hu), Lamp1 (Santa Cruz Biotechnology, sc-20011, FM, 1:100), Lamp2 (Abcam, ab25631, clone H4B4, FM, 1:200, Hu), PDIA3 (Sigma-Aldrich, AMAB90988, clone CL2444, lot 02879, FM, 1:200, Hu), MHCII-APC/Cyanine7 (BioLegend, 107628, clone M5/114.12.2, lot B370049, FCy, 1:100, Ms), Rcas1 (Cell Signaling, 12290S, clone D2B6N, lot 1, FM, 1:200, Hu), SLC7A11/xCT (Cell Signaling, 12691S, clone D2M7A, lot 5, WB, 1:1000, Hu, siRNA validated by us), TfR1-APC-AF750 (Beckman Coulter, A89313, clone YDJ1.2.2, lot 200060, FCy, 1:100, Hu), TfR1-PE (BioLegend, 334106, clone CY1G4, lot B364886, FCy, 1:100, Hu), γ-tubulin (Sigma-Aldrich, T5326, clone GTU-88, lot 0000140390, WB, 1:1000, Hu, validation by manufacturer). Secondary antibodies: Alexa Fluor 647 anti-mouse (Abcam, ab150115, tissue labelling, 1:500, Ms), Alexa Fluor 647 anti-mouse (Invitrogen, A21237, lot 1485202, FM, 1:1000, Hu), Alexa Fluor 647 anti-rabbit (Invitrogen, A21246, lot 2714437, FM, 1:1000, Hu), donkey anti-rabbit IgG-h+l HRP-conjugated (Bethyl Laboratories, A120–108P lot 13, WB, 1:10000, Hu), goat anti-mouse IgG-h+l HRP-conjugated (Bethyl Laboratories, A90–116P, lot 39, WB, 1:10000, Hu), goat anti-rat IgG-h+l HRP-conjugated (Invitrogen, 31470, WB, 1:10000, Hu).

#### Cell culture.

Dissociated human and mouse tumour cells and 4T1 cells were cultured in RPMI 1640 supplemented with GlutaMAX (Gibco, 61870010), 10% foetal bovine serum (FBS, Eurobio Scientific, CVFSVF00–01). HT-1080 cells were cultured in Dulbecco’s Modified Eagle Medium GlutaMAX (DMEM, Gibco, 61965059) supplemented with 10% FBS (Gibco, 10270–106) and penicillin/streptomycin (BioWhittaker/Lonza, DE17–602E). FC1242 and FC1245 murine pancreatic cancer cells, 4a cells and human pancreatic hMIA-2D cells were a generous gift from the Tuveson laboratory (CSHL) and were cultured in DMEM supplemented with 10% FBS and penicillin/streptomycin. Primary human pancreatic PDAC090T, PDAC053T, PDAC211T and PDAC030T cells were grown in serum-free ductal medium: DMEM/F12 supplemented with 0.61g/500μL nicotinamide (Sigma-Aldrich, 3376), 2.50g/ 500μL glucose (Sigma-Aldrich, G6152), 1:200 ITS+ (Corning, 354352), 1:20 Nu-serum IV (Corning, 355104), 100 ng/μL. cholera toxin, 1 μM dexamethasone (Sigma-Aldrich, D4902), 50 nM 3,3’,5-triiodo-L-thyronine (Sigma-Aldrich, T6397) and penicillin/streptomycin.

#### Dissociation of human and murine tumour samples:

Tumour samples were collected from patients after surgery. Tumour samples correspond to pancreatic ductal adenocarcinoma (PDAC), undifferentiated pleomorphic sarcoma (UPS), liposarcoma, angiosarcoma, epithelioid sarcoma and PDAC liver metastasis. Tumours were dissociated using the human tumour dissociation kit (Miltenyi, 130-095-929) according to the manufacturer’s protocol. In brief, tumours were cut into small pieces of 1–5 mm, put in presence of the enzyme mix in RPMI and dissociated on the gentleMACS Octo Dissociator with Heaters (Miltenyi) with the appropriate gentleMACS program (37C_h_TDK). Per tumour sample, a total volume of 9.4 μL of medium was used with the corresponding enzyme concentration according to the manufacturer’s protocol. Mouse tumour samples were dissociated using the mouse tumour dissociation kit (Miltenyi, 130-096-730) according to the manufacturer’s protocol. Tumours were cut into small pieces of 1–5 mm, put in presence of the enzyme mix in RPMI and dissociated on the gentleMACS Octo Dissociator with Heaters (Miltenyi) with the appropriate gentleMACS program (37C_m_TDK).

Subsequently, the dissociated tumour suspension was applied to a MACS SmartStrainer (30 μm) (Miltenyi). Samples were diluted with 1× PBS (Phosphate-buffered saline) and centrifuged at 300× g. The cell pellet was resuspended in RPMI (10%FBS, penicillin/streptomycin) and cells were counted using an automated cell counter (Entek)

#### Establishment of xenograft derived primary cell cultures (XDPCC).

XDPCC models were originally derived from PDX patient models. The PDX fragments designated for cell culture were processed in a biosafety chamber. After fine mincing they were treated with collagenase type V (Sigma-Aldrich, C9263) and trypsin/EDTA (Gibco, 25200–056) and were suspended in Dulbecco’s modified Eagle’s medium supplemented with 1% w/w penicillin/streptomycin and 10% FBS. After centrifugation, cells were resuspended in serum-free ductal media adapted from previous protocols^52^ at 37 °C in a 5% CO_2_ incubator. Amplified cells were stored in liquid nitrogen. Cells were weaned from antibiotics for more than 48 h before testing. This protocol was used to establish the cells designated as PDAC053T, PDAC090T, PDAC211T and PDAC030T.

#### Establishment of xenograft derived pancreatic organoids (XDPO).

XDPO models were originally derived from PDX patient models. Xenografts were split into several small pieces and processed in a biosafety chamber and after a fine mincing they were treated with the tumour dissociation kit. Undigested pellets were digested with accutase at 37 °C for 30 min. The pancreatic tissue slurry was transferred into a tissue strainer 100 μm and was placed into 12-well plate coated with 150 μL GFR matrigel (Corning). The samples cultured with pancreatic organoid feeding media (POFM) consisted of advanced DMEM/F12 supplemented with 10 mM HEPES (Thermo Fisher Scientific, 15630056), 1× Glutamax (Thermo Fisher Scientific, 35050087), penicillin/streptomycin, 100 ng/μL animal-free recombinant human FGF10 (Thermo Fisher Scientific, 500-P151G-50UG), 50 ng/μL animal-free recombinant human EGF (Thermo Fisher Scientific, AF-100-15-1MG), 100 ng/μL recombinant human noggin (Biotechne, 6057-NG), Wnt3a-conditioned medium (30% v/v); RSPO1-conditioned medium (10% v/v), 10 nM human gastrin 1 (Sigma-Aldrich, SCP0152) 10 mM nicotinamide (Sigma-Aldrich, N0636), 1.25 mM N-acetylcysteine (Sigma-Aldrich, A9165), 1× B27 (Thermo Fisher Scientific, 17504001), 500 nM A83–01 (Tocris, 2939/10) and 10.5 μM Y27632 (Tocris, 1254/1). The plates were incubated at 37 °C in a 5% CO_2_ incubator, and the media were changed every 3 or 4 days. This procedure was used to generate the XDPOs PDAC009T, PDAC003T, PDAC117T and PDAC372T.

#### Chemosensitivity profiling of XDPO and XDPCC.

For chemosensitivity profiling, XDPO were plated into 96-well plates and then subjected to incrementally increasing concentrations of drugs. Cell viability was measured 72 h after treatment using CellTiter-Glo 3D (Promega, G9683). Doubling times (DT) of XDPO viability for untreated control conditions were calculated on days 0 and 3. The ratio of day 3 over day 0 corresponds to the replication rate (RR) of the cells at 72 h. Doubling time was calculated with the formula 72 × 2/RR. Fluorescence and luminescence values were quantified using the plate reader Tristar LB941 (Berthold Technologies). Each experiment was performed at least 3 times with at least 3 replicates.

#### Cell death and viability assays.

Cell death assay with Annexin-V (A) & Propidium Iodide (PI): PDAC-053T cells were seeded on 6-well plates at the density of 2× 10^5^ cells/well. Baf-A1 (75 nM) was added 7 h prior to the experiment. RSL3 (Sigma-Aldrich, SμL2234, 0.1, 0.5, 2, 10 μM) was added along with Lip-1 (1 μM), cLip-1 (1 μM), metcLip-1 (1 μM) or alcLip-1 (1 μM) on the following day, and after 24 h, the media was recovered and cells were trypsinised. Cells were harvested, pelleted along with the media recovered, washed with 1× PBS, and 100 μL of 1× Annexin-V binding buffer containing Annexin-V and PI according to the manufacturer’s protocol (Annexin-V flow cytometry kit, Thermo Fisher Scientific, V13242). 1× PBS buffer containing 10% FBS and EDTA (0.1% v/v) was added for flow cytometry. Flow cytometry was run on an AttuneTM NxT Flow Cytometer and analysed on FlowJo. Lactate dehydrogenase release was measured using a Cytotoxicity detection kit (Sigma-Aldrich, 11644793001) according to the manufacturer’s protocol in a 96-well format. Cell viability was assessed using a CellTiterGlo 2.0 (Promega, G9241) or CellTiter blue (Promega, G8081) kit according to the manufacturer’s protocol in a 96-well format. In brief, 4000 HT-1080 cells were seeded per well in clear-bottom and darkened 96-well plates (Greiner, 655090, lot E23063EG) 24 h prior to the experiment. Cells were then pre-treated for 2 h with Lip-1 (10 μM), cLip-1 (10 μM), ferrostatin-1 (Fer-1, SμL0583, 10 μM), deferoxamine (DFO, Sigma-Aldrich, D9533, 100 μM), deferasirox (DFX, Cayman chemical, 16753, 10 μM), deferiprone (Def, Sigma-Aldrich, Y0001976, 100 μM), tocopherol (Toc, Sigma-Aldrich, PHR1031, 100 μM), Vitamin K3 (Sigma-Aldrich, M5625–25G, 10 μM), Z-VAD-FMK (Enzo Life Sciences, ALX-260–020-M005, 50 μM) or Necrostatin-1 (Nec-1, Sigma-Adrich, N9037, 20 μM). Subsequently fentomycin (10 μM, 6 h) was added. Samples were processed detailed in the manufacturer’s protocols and data recorded on a SpectraMax ID3 plate reader (Molecular Devices). For standard-of-care cell survival measurements, cells were plated at 2000 cells per well 24 h prior to the experiment. Cells were incubated with serial dilutions of fentomycin, irinotecan (Sigma-Aldrich, I1406), 5-FU (Alfa Aesar, A13456–06) or oxaliplatin (Biotechne, 2623) for 72 h. Cell viability was assessed using a CellTiter blue assay and data recorded on a SpectraMax ID3 plate reader (Molecular Devices). For MTT assays, cells were plated in a 96-well plate, incubated for 24 h, and then pre-treated with Lip-1 (1uM) for 10 min prior to treatment with vehicle control or fentomycin for 24 h. After 24 h, media was carefully removed and 50 μL of serum-free media and 50 μL of MTT solution (Cayman Chemical, 21795) were added per well. The plate was incubated at 37 °C for 3 h. After incubation, the solution was removed and 100 μL of DMSO was added per well. The plate was covered and shaken on an orbital shaker for 15 min prior to reading absorbance on a plate reader (OD=590). For RSL3 treatment, Pfa1 cells were seeded onto 96-well plates (2000 cells per well) and cultured overnight. The following day, cells were cotreated with RSL3 (500 nM) and indicated small molecules in serial dilution. For 4-OH-TAM treatment, Pfa1 cells were seeded onto 96-well plates (500 cells per well) with 4-OH-TAM (1 μM) and a dilution series of the indicated compounds. After 24 h (for RSL3) or 72 h (for 4-OH TAM) incubation, cell viability was assessed using resazurin as a viability indicator. Fluorescence intensity was measured at Ex/Em = 540/590 nm using a SpectraMax iD5 microplate reader with SoftMax Pro v7 software (Molecular Devices) after 4 h of incubation in standard cell culture medium containing 0.004% resazurin.

#### cLip-1 treatment in vivo.

Mice were kept under standard conditions with water and food *ad libitum* and in a controlled environment (22 ± 2 °C, 55 ± 5% humidity, 12 h light/dark cycle). For animal studies, C57BL6/J mice were randomised into separate cages. 12 to 24-week-old sex-matched mice were used for all experiments. For the survival cohort study, Rosa26-CreERT2;*Gpx4*^f/f^ mice were intraperitoneally treated with tamoxifen (Sigma-Aldrich, T2859, 2 mg/day dissolved in Myglyol 812) on day 0 and day 1 for deletion of *Gpx4* in the whole body except in the brain^4^. From day 2, cLip-1 (10 mg/kg/day dissolved in 1x PBS containing 20% PEG400 and 5% Solutol HS15) or vehicle was intraperitoneally administered to the mice each day until the completion of the survival study. For histochemistry analyses with click chemistry, Rosa26-CreERT2;*Gpx4*^f/f^ mice treated with intraperitoneal tamoxifen injection (2 mg/day on day 0 and day 1) were used. On day 7, cLip-1 (10 and 100 mg/kg) or vehicle was intraperitoneally injected to the mice. One h after the injection, mice were euthanised and the kidney and liver samples were collected.

#### Isolation of blood, serum and lymphatic fluid from mice.

Balb/C mice (25-week-old adult female mice) were purchased from Charles River and grow in CRCM animal core facility. Mice were housed under sterile conditions with sterilised food and water provided *ad libitum* and maintained on a 12 h light/dark cycle.Mice were not subjected to any procedures prior to the lymph, blood and serum samples collection. *Lymph sample collection:*Thirty min before the beginning of the experiment, buprenorphine (Buprecare), an analgesic, was administered by intraperitoneal injection (0.5 mg/kg). Mice were euthanised by intraperitoneal injection of a ketamine/xylazine combination (ketamine 100 mg/kg (Imalgène)/xylazine 10 mg/kg (Rompum); 20 μl/g). After cutaneous and peritoneal incisions, the lymph has been collected in the intestinal lymph trunk^53^ with a glass capillary. The lymph collection was placed in cryotubes, frozen at −20 °C and stocked at −80 °C. *Blood and serum sample collection:* After lymph collection, we performed a terminal cardiac puncture (23G needle with 1 to 2 μL syringe) with thoracotomy to collect a large volume of blood without anticoagulants. 100 to 200 μL of blood sample were placed in a vial, frozen at −20 °C and stocked at −80 °C. The rest of the blood collection was left to stand at room temperature for 30 min, then centrifuged at 2000x g for 15 min. The supernatant (serum) was collected in a vial, frozen at −20 °C and stored at −80 °C.

#### Intranodal murine metastasis models and fentomycin treatment.

Murine breast cancer cells (4T1) were transplanted into 6 to 8-week-old female Balb/c mice (syngeneic with the 4T1 model). To perform injections into lymph nodes, the lymphatics were first traced by injecting 2% Evans Blue dye (Sigma-Aldrich, E2129,) into the foot pedal 5 min before performing intranodal injections. After injecting Evans Blue dye, the mice were anesthetised using isoflurane and a small (5–10 mm) incision was made in the region of the right popliteal lymph node. The lymph node was located based on Evans Blue staining, immobilised with forceps, and 20000 cells (Experiment 1) to 10000 cells (Experiments 2 and 3) suspended in 1x PBS were injected in a volume of 10 μL into the popliteal lymph node using a 27 G Hamilton syringe. Injection into the lymph node was confirmed by visible swelling of the lymph node. The incision was closed using surgical glue (3M VetBond Tissue Adhesive, 1469SB,) and the mice were closely monitored for signs of pain or distress. Once tumours were palpable in at least 75% of the mice (~1 week after injection), 10 μL of volume of fentomycin (0.003 mg per animal every-other-day) of vehicle delivered intralymphatically into the tumour-bearing lymph node every other day until the experimental endpoint. Intranodal tumour diameters were measured thrice weekly with calipers until any tumour in the mouse cohort reached 2.0 cm in its largest diameter which was the pre-determined experimental endpoint for these experiments. At that point, all mice in the cohort were killed, per approved protocol, for analysis of intranodal tumour diameter, tumour mass and mouse. Tumour samples were frozen in 10% DMSO in FBS (1 °C/ min until −80 °C) for subsequent cellular analyses. No formal randomisation techniques were used. However, animals were allocated randoμLy to treatment groups and specimens were processed in an arbitrary order. For all experiments, the maximum permitted tumour diameter was 2.0 cm and this limit was not exceeded in any experiment. For all experiments, mice were kept on a normal Chow diet and fed ad-libitum.

#### Fluorescence microscopy.

Cells were plated on coverslips and treated as indicated. BODIPY 665/676 (Thermo Fisher Scientific, B3932, 10 μM, 45 min), LysoTracker Deep red (Thermo Fisher Scientific, L12492, 100 nM, 45 min), SQSS (in-house, 50 nM, 24 h) and 1-Red (in-house, 100 nM, 1 h before fixation) were added to live cells before fixation. For fentomycin and marmycin A treatments, cells were treated at the indicated temperature with 1 μM compound for 1 h. BacMam transduced cells (see transduction section) were treated with BODIPY 665/676 16 h after transduction. Cells were then washed three times with 1× PBS, fixed with 2% paraformaldehyde in 1× PBS for 12 min and then washed three times with 1× PBS. For antibody staining, cells were then permeabilised with 0.1% Triton X-100 in 1× PBS for 5 min and washed three times with 1× PBS. Subsequently, cells were blocked in 2% bovine serum albumin/BSA, 0.2% Tween-20/1× PBS (blocking buffer) for 20 min at room temperature. Cells were incubated with the relevant antibody in blocking buffer for 1 h at room temperature, washed three times with 1× PBS and were incubated with secondary antibodies for 1 h. Finally, coverslips were washed three times with 1× PBS and mounted using VECTASHIELD containing DAPI (Vector Laboratories, H-1200–10). BODIPY 665/676 treated cells were fixed using ice cold reagents and placed at 4 °C immediately after mounting on cover slips and imaged immediately. Fluorescence images were acquired using a Deltavision real-time microscope (Applied Precision) or a thunder microscope (Leica). 40×/1.4NA, 60×/1.4NA and 100×/1.4NA objectives were used for acquisitions and all images were acquired as z-stacks. Images were deconvoluted with SoftWorx (Ratio conservative - 15 iterations, Applied Precision) and processed with FIJI 2.0.0-rc-69/1.52n. Images were taken in black and white and colouring was applied with FIJI. Fluorescence intensity is displayed as arbitrary units (AU) and is not comparable between different panels. Colocalisation quantification was calculated using FIJI 2.0.0-rc-69/1.52n. Nuclei were detected using DAPI or Hoechst fluorescence as indicated.

#### Small molecule labelling using click chemistry.

##### In-cell click labelling:

Cells on coverslips were treated as indicated with cDFO (in-house, 100 μM, 15 min), cLip-1 (in-house, 1 μM or 10 μM, 1 h), metcLip-1 (in-house, 10 μM, 1 h), alcLip-1 (in-house, 10 μM, 1 h), then fixed and permeabilised as indicated in the fluorescence microscopy paragraph. LysoTracker Deep Red was added to live cells for 45 min before fixation. The click reaction cocktail was prepared using the Click-iT EdU Imaging kit (Invitrogen, C10337) according to the manufacturer’s protocol. In a typical experiment we mixed 50 μL of 10× Click-iT reaction buffer with 20 μL of CuSO_4_ solution, 1 μL Alexa-Fluor-azide, 50 μL reaction buffer additive (sodium ascorbate (Asc)) and 379 μL ultrapure water to reach a final volume of 500 μL. Coverslips were incubated with the click reaction cocktail in the dark at room temperature for 30 min, then washed three times with 1× PBS. Immunofluorescence was then performed as described in the fluorescence microscopy section. *Click labelling in tissues:* The kidney and liver tissue samples collected from the cLip-1-treated mice were fixed in 4% paraformaldehyde in 1× PBS overnight at 4 °C. Fixed tissues were incubated in 10% sucrose in 1× PBS for 30 min and then incubated in 20% sucrose in 1× PBS at 4 °C for 4 h, followed by embedding in OCT mounting compound (TissueTek, Sakura) on dry ice and storage at −80 °C. Frozen tissues were cut into 5-μm-thick sections using a Cryostat Microm HM 560 (Thermo Fisher Scientific) at −30 °C. Tissue sections were postfixed with 1% paraformaldehyde in 1× PBS for 10 min and subsequently incubated with 100% acetone for 10 min at −20 °C. Sections were incubated in blocking solution (1× PBS containing 3% BSA and 0.2% Triton X-100) for 30 min. The specimens were labelled using a click reaction as described above. For co-staining with a lysosome marker, the click reaction labelled specimens were incubated with anti-Lamp1 antibody diluted in 1× PBS containing 10% normal goat serum overnight at 4 °C. The next day, sections were incubated with secondary antibody in 1× PBS containing 1% BSA and 0.3% Triton X-100 for 2 h at room temperature. Nuclei were visualised with Hoechst 33342 staining, and slides were mounted in Aqua/PolyMount (Polysciences). Images were acquired using a fluorescence microscope (DS-Qi2, Nikon) or confocal microscope (LSM880, Carl Zeiss) and the corresponding appropriate filter sets for fluorophores.

#### Western blotting.

Cells were treated as indicated and then washed with 1× PBS. Proteins were solubilised in 2× LaemμLi buffer containing benzonase (VWR, 70664–3, 1:100). Extracts were incubated at 37 °C for 1 h, heated at 94 °C for 10 min and quantified using a NanoDrop 2000 spectrophotometer (Thermo Fisher Scientific). Protein lysates were resolved by SDS-PAGE electrophoresis (Invitrogen sure-lock system and Nu-PAGE 4–12% Bis-Tris precast gels). In a typical experiment, 10–20 μg of total protein extract were loaded per lane in 2× LaemμLi buffer containing bromophenol blue. On each gel a size marker was run: 3 μL PageRuler (Thermo Fisher Scientific, 26616) or 3 μL PageRuler plus (Thermo Fisher Scientific, 26620) and 17 μL 2× LaemμLi buffer). Proteins were then transferred onto nitrocellulose membranes (Amersham Protran 0.45 μm) using a Trans-Blot SD semi-dry electrophoretic transfer cell (Bio-Rad) using 1× NuPage transfer buffer (Invitrogen, NP00061) with 10% methanol. Membranes were blocked with 5% non-fat skimmed milk powder (Régilait) in 0.1% Tween-20/1 × PBS for 20 min. Membranes were cut at the appropriate marker size to allow for the probing of several antibodies on the same membrane. Blots were then probed with the relevant primary antibodies in 5% BSA, 0.1% Tween-20/1× PBS or in 5% non-fat skimmed milk powder in 0.1% Tween-20/1× PBS at 4 °C overnight with gentle motion in a hand-sealed transparent plastic bag. Membranes were washed with 0.1% Tween-20/1× PBS three times and incubated with horseradish peroxidase conjugated secondary antibodies (Jackson Laboratories) in 5% non-fat skimmed milk powder, 0.1% Tween-20/1× PBS for 1 h at room temperature and washed three times with 0.1% Tween-20/1× PBS. Antigens were detected using the SuperSignal West Pico PLUS (Thermo Fisher Scientific, 34580) and SuperSignal West Femto (Thermo Fisher Scientific, 34096) chemiluminescent detection kits. Signals were recorded using a Fusion Solo S Imaging System (Vilber). γ-Tubulin served as loading control on the same gels. Band quantifications were performed with FIJI 2.0.0-rc-69/1.52n using pixel intensity normalised against the signal of γ-tubulin. All full scans of blots are displayed in the Supplementary Information.

#### Transduction.

PDAC053T cells were seeded at 200000 cells per well 24 h prior to the experiment in 1 μL of medium. Cells were then transduced with CellLight Lysosomes-GFP, BacMam 2.0 (Thermo Fisher Scientific, C10596), CellLight ER-GFP, BacMam 2.0 (Thermo Fisher Scientific, C10590), CellLight Mitochondria-GFP, BacMam 2.0 (Thermo Fisher Scientific, C10600) according to the manufacturer’s procedure. In brief, 70 μL of BacMam reagent was added to the medium and mixed. Cells were incubated for 16 h.

#### Flow cytometry.

Cells were washed with ice-cold 1× PBS. For antibody staining, cells were incubated with Fc block (Human TruStain FcX, Biolegend, 422302, 1:20) for 15 min, then incubated with antibodies in ice cold 10% FBS, 1× PBS, 2 mM EDTA for 20 min at 4 °C and then washed with 1× PBS and resuspended in 10% FBS, 1× PBS, 2 mM EDTA before analysis using a flow cytometer. For BODIPY-C11 581/591 staining, live cells were incubated with BODIPY-C11 581/591 (Thermo Fisher Scientific, D3861, 1 μM, 45 min) before fixation. RPE probe flow cytometry: PDAC-053T cells were seeded in 6-well plates at the density of 2× 10^5^ cells/well. On the following day, compounds were added as indicated in the figure for 1 h; ferric ammonium citrate (FAC, Sigma-Aldrich, F5879, 100 μg/μL), Lip-1 (10 μM), hydroxychloroquine (HCQ, Sigma-Aldrich, H0915, 100 μM), Bafilomycin A1 (Baf-A1, Sigma-Aldrich, B1793, 75 nM). After 30 min of treatment with compounds, RPE probe (in-house, 40 μM) was added for 30 min. The media was removed and cells were washed with 1× PBS once before trypsinisation. Cells were harvested, pelleted, washed with 1× PBS and finally 250 μL of 1× PBS buffer containing 10% FBS and EDTA (0.1% v/v), was added for flow cytometry. Data were recorded on a BD LSR Fortessa X-20. BODIPY-C11 581/591 flow cytometry on PDAC-053T cells: PDAC-053T cells were seeded in 6-well plates at the density of 2× 10^5^ cells/well. On the next day, cells were treated with Baf-A1 (75 nM) and HCQ (10 μM) for 7 h before adding RSL3 (200 nM). Then after 16 h, cells were treated with BODIPY-C11 581/591 (4 μM) for an additional 1 h. The media was removed and cells were washed with 1× PBS twice before trypsinisation. Cells were harvested, pelleted, washed with 1× PBS and finally 250 μL of 1× PBS buffer containing 10% FBS and EDTA (0.1% v/v), was added for flow cytometry. Data were recorded on an AttuneTM NxT Flow Cytometer (Thermo Fisher Scientific). For flow cytometry analyses of lysosomal GSH and lysosomal hydroxyl radicals: Cells were incubated with SQSS (100 nM, 24 h) or 1-Red. HT-1080 cells were incubated with RSL3 (1 μM), μL210 (10 μM, Sigma-Aldrich, SμL0521), FIN56 (5 μM, Sigma-Adrich, SμL1740), Buthionine sulfoximine (BSO, 10 μM, Sigma-Aldrich, B2515) or erastin (10 μM, Sigma-Aldrich, 329600) for the indicated time points. Data were recorded on an AttuneTM NxT Flow Cytometer (Thermo Fisher Scientific). For flow cytometry analyses of lysosomal iron content typically 200000 dissociated human tissue cells were incubated in media (RPMI 1610, 10% FBS, penicillin/streptomycin) with RhoNox-M (in-house, 1 mM, 1 h). Lysosomal iron content of human tumour samples and healthy adjacent tissues was analysed with the following antibody and stain panel: DAPI (0.1 μg/μL), CD3-BV510 (BioLegend, 317332), CD31-PE-Cy7 (BioLegend, 303118), CD44-AF647 (Novus Biologicals, NB500–481AF647), CD45-BV785 (BioLegend, 304048), CD163-PerCP/Cyanine5.5 (BioLegend, 326512), FAP-AF700 (R&D Systems, FAB3715N), TfR1-APC-AF750 (Beckman Coulter, A89313). The live tumour cells corresponded to DAPI^neg^/CD45^neg^/CD31^neg^/FAP^neg^ cells. Data were recorded on a BD LSRFortessa X-20. For flow cytometry analyses of CD44 in fentomycin treated tumour samples, typically 200000 dissociated tumour cells were incubated in media (RPMI 1610, 10% FBS, penicillin/streptomycin) with 1 μM fentomycin for 24 h. Cells were pre-treated with 1 μM Lip-1, 1 μM cLip-1, 100 μM Toc or 100 μM Def for 2 h. Toc was kept pure under inert atmosphere and a fresh stock solution was prepared throughout the study before each experiment. The following antibody and stain panel was used for subsequent flow analysis: SYTOX blue (Thermo Fisher Scientific, S34857, 1 μM), CD31-BV605 (BioLegend, 303122), CD45-BV510 (BioLegend, 368526, lot B373428), CD44-AF647 (Novus Biologicals, NB500–481AF647), TfR1-PE (BioLegend, 334106), FAP-AF750 (Novus Biologicals, FAP3715S). The live tumour cells corresponded to SYTOX blue^neg^/CD45^neg^/CD31^neg^/FAP^neg^ cells. Data were recorded on an AttuneTM NxT Flow Cytometer (Thermo Fisher Scientific). For flow cytometry analyses of CD44 levels in mouse 4T1 tumours, typically 200000 dissociated tumour cells were used per condition. Freshly dissociated cells were stained using the following antibody and stain panel: SYTOX blue (Thermo Fisher Scientific, S34857, 1 μM), CD31-BV605 (BioLegend, 102427), CD44-AF647 (BioLegend, 103018), CD45-BV510 (BioLegend, 103138), MHCII-APC/Cyanine7 (BioLegend, 107628). The live tumour cells corresponded to SYTOX blue^neg^/CD45^neg^/CD31^neg^/MCH II^pos^ cells. Data were recorded on an AttuneTM NxT Flow Cytometer (Thermo Fisher Scientific). All data were analysed with FlowJo software v. 10.10.0.

#### Fluorescence-activated cell sorting.

Sorting of human cells was performed using the following antibodies: CD31-PE-Cy7 (BioLegend, 303118), CD44-AF647 (Novus Biologicals, NB500–481AF647), CD45-BV785 (BioLegend, 304048). The sorted cells corresponded to CD45^neg^/CD31^neg^/CD44^pos^ cells and CD45^neg^/CD31^neg^/CD44^neg^ cells and were isolated on a FACSAria Fusion (BD). ICP-MS experiments were conducted in CD44^pos^ and CD44^neg^ tumour cells as described in the ICP-MS section. Sorting of murine cells was performed using the following antibodies: CD44-AF647 (Biolegend, 103018), MHCII-APC/Cyanine7 (BioLegend, 107628). The sorted cells corresponded to MHCII^pos^/CD44^pos^ cells and MHCII^pos^/CD44^neg^ cells. Sorted cells were centrifuged at 300× g and cell pellets were processed for subsequent applications.

#### Inductively coupled plasma mass spectrometry (ICP-MS).

Glass vials equipped with teflon septa were cleaned with nitric acid 65% (VWR, Suprapur, 1.00441.0250), washed with ultrapure water (Sigma-Aldrich, 1012620500) and dried. Cells were harvested and washed twice with 1× PBS. Cells were then counted using an automated cell counter (Entek) and transferred in 200 μL 1× PBS or ultrapure water to the cleaned glass vials. The same volume of 1× PBS or ultrapure water was transferred into separate vials for the background subtraction, at least in duplicate per experiment. For tissue samples, a small piece of about 1 mm^3^ was transferred into a clean pre-weighed vial. Samples were lyophilised using a freeze dryer (CHRIST, 2–4 LDplus). Vials with tissue samples were weighed subsequently to determine the tissue dry weight. Samples were then mixed with nitric acid 65% and heated at 80 °C overnight in the same glass vials closed with a lid carrying a teflon septum. Samples were then cooled to room temperature and diluted with ultrapure water to a final concentration of 0.475 N nitric acid and transferred to metal-free centrifuge vials (VWR, 89049–172) for subsequent mass spectrometry analyses. Amounts of metals were measured using an Agilent 7900 ICP-QMS in low-resolution mode, taking natural isotope distribution into account. Sample introduction was achieved with a micro-nebulizer (MicroMist, 0.2 μL/min) through a Scott spray chamber. Isotopes were measured using a collision-reaction interface with helium gas (5 μL/min) to remove polyatomic interferences. Scandium and indium internal standards were injected after inline mixing with the samples to control the absence of signal drift and matrix effects. A mix of certified standards was measured at concentrations spanning those of the samples to convert count measurements to concentrations in the solution. Values were normalised against cell number or tissue dry weight.

#### Lipidomics.

For comparison of ferroptosis inducers, HT1080 cells were treated with fentomycin (1 μM), erastin (10 μM), RSL3 (100 nM) or iFSP1 (10 μM, Sigma-Aldrich, SμL2749). For co-treatment with ferroptosis inhibitors, HT1080 cells were pre-treated with Toc (100 μM), Def (100 μM) or Lip-1 (10 μM) for 2 h, and then co-treated with fentomycin (1 μM) for 24 h. Dissociated human tumour samples were pre-treated with 100 μM Toc and then co-treated with 1 μM fentomycin for 24 h. Dissociated mouse tumour samples were counted and processed directly after dissociation. Cells were subsequently washed with 1× PBS and then with 150 mM ammonium bicarbonate. Cells were then resuspended in 150 mM ammonium bicarbonate and centrifuged at 300× g for 5 min. The supernatant was removed and cells were resuspended in 1 μL of 150 mM ammonium bicarbonate. The solutions were centrifuged at 12000 rpm for 10 min and the supernatant was removed. 200 μL of 150 mM sodium bicarbonate was added to the pellet and samples were flash frozen in liquid nitrogen. Lipidomics analysis was performed on the same day for all technical and biological replicates for a given dataset. For lipidomics analysis, the 200 μL cell lysates were spiked with 1.4 μL of internal standard lipid mixture containing 300 pmol of PC 17:0–17:0, 50 pmol of PE 17:0–17:0, 50 pmol of PI 16:0–16:0, 50 pmol of PS 17:0–17:0, and 30 pmol of PA 17:0–17:0, 30 pmol of LPC 12:0, 30 pmol of LPE 17:1, 30 pmol of LPS 17:1 and 30 pmol of LPA 17:0 and subjected to lipid extraction at 4 °C as previously described^54^. Briefly, the sample was extracted with 1 μL of chloroform:methanol (10:1) for 2 h at 4 °C with vigorous shaking in a thermomixer (1000 rpm). The lower organic phase was collected and dried in a Speedvac vacuum concentrator. The remaining aqueous phase was re-extracted with 1 μL of chloroform:methanol (2:1) for 1 h at the same temperature and shaking conditions. The lower organic phase was collected and evaporated in a SpeedVac vacuum concentrator. Lipid extracts were dissolved in 100 μL of infusion mixture consisting of 7.5 mM ammonium acetate dissolved in propanol:chloroform:methanol [4:1:2 (v/v)]. Samples were analysed by direct infusion in a QExactive mass spectrometer (Thermo Fisher Scientific) equipped with a TriVersa NanoMate ion source (Advion Biosciences). 5 μL of sample were infused with gas pressure and voltage set to 1.25 psi and 0.95 kV, respectively. PC and PC_Ox_ were detected in the 10:1 extract, by positive ion mode FTMS as protonated adducts by scanning m/z= 580–1000 Da, at R_m/z=200_=280 000 with lock mass activated at a common background (m/z=680.48022) for 30 seconds. Every scan is the average of 2 micro-scans, automatic gain control (AGC) was set to 10^6^ and maximum ion injection time (IT) was set to 50 ms. PE, PEO, PE_Ox_ and LPE were detected as deprotonated adducts and LPC were detected as acetate adducts in the 10:1 extract, by negative ion mode FTMS by scanning m/z= 420–1050 Da, at R_m/z=200_ =280 000 with lock mass activated at a common background (m/z=529.46262) for 30 seconds. Every scan is the average of 2 micro-scans, automatic gain control (AGC) was set to 10^6^ and maximum ion injection time (IT) was set to 50 ms. The following abbreviations are used: PC = phosphatidylcholine, PE = phosphatidylethanolamine, PS = phosphatidylserine, PI = phosphatidylinositol, _ox_ = oxidised. LPC = lysophosphatidylcholine, LPE = lysophosphatidylethanolamine, LPS = lysophosphatidylserine, LPI = lysophosphatidylinositol. PI, PI_Ox_, PS, PS_Ox_, LPI, and LPS were detected in the 2:1 extract, by negative ion mode FTMS as deprotonated ions by scanning m/z= 400–1100 Da, at R_m/z=200_=280 000 with lock mass activated at a common background (m/z=529.46262) for 30 seconds. Every scan is the average of 2 micro-scans, automatic gain control (AGC) was set to 10^6^ and maximum ion IT was set to 50 ms. All data was acquired in centroid mode. All lipidomics data were analysed with the lipid identification software, LipidXplorer (http://genomebiology.com/2011/12/1/R8). Tolerance for MS and identification was set to 2 ppm. Data post-processing and normalisation to internal standards were done in Excel (Microsoft).

#### Glycerol quantification.

HT1080 cells were treated with 1 or 2 μM fentomycin for 24 h. Glycerol was quantified using the Glycerol-glo assay (Promega, J3150) according to the manufacturer’s protocol. In brief, the assay was performed in a 96-well plate and 4000 cells were plated per well 24 h prior to the experiment. A standard curve was prepared for each biological experiment and three technical replicates were performed for each condition and each biological replicate. Luminescence signals were recorded using a SpectraMax ID3 plate reader (Molecular Devices). Data were exported and analysed using Excel (Microsoft) and PRISM software.

#### Software for illustrations.

Illustrations were created using FIJI 2.0.0-rc-69/1.52n, Prism 10.0.3 and Adobe Illustrator 26.0.2 and biorender.com. Biorender.com was used for Fig. 2b and Fig. 4.

#### Quantification, statistical analysis and reproducibility.

Results are presented as mean values ± standard error of the mean (s.e.m.) or standard deviation (s.d.) as indicated. Box plots: boxes represent interquartile range and median, and whiskers indicate the minimum and maximum values. Prism 10.0.3 software was used to calculate *P* values using a two-sided Mann-Whitney test, two-sided unpaired t-test, Kruskal-Wallis test with Dunn’s post-test, 2-way ANOVA or Mantel-Cox log-rank test as indicated. Prism 10.0.3 software was used to generate graphical representations of quantifications unless stated otherwise. Exact *P* values are indicated in the figures. Sample sizes (*n*) are indicated in the figure legends.
